# Prevention of Chilling Injury in Pomegranates Revisited: Pre- and Post-Harvest Factors, Mode of Actions, and Technologies Involved

**DOI:** 10.3390/foods12071462

**Published:** 2023-03-29

**Authors:** Mahshad Maghoumi, Maria Luisa Amodio, Luis Cisneros-Zevallos, Giancarlo Colelli

**Affiliations:** 1 Dipartimento di Scienze Agrarie, Degli Alimenti e dell’Ambiente, Università di Foggia, Via Napoli 25, 71122 Foggia, Italy; mahshad.maghoumi@unifg.it (M.M.);; 2 Department of Horticultural Sciences, Texas A&M University, College Station, TX 77843, USA

**Keywords:** pomegranate, browning, oxidative stress, long-term storage, chilling injury, polyphenols, postharvest treatments

## Abstract

The storage life of pomegranate fruit (*Punica granatum* L.) is limited by decay, chilling injury, weight loss, and husk scald. In particular, chilling injury (CI) limits pomegranate long-term storage at chilling temperatures. CI manifests as skin browning that expands randomly with surface spots, albedo brown discoloration, and changes in aril colors from red to brown discoloration during handling or storage (6–8 weeks) at <5–7 °C. Since CI symptoms affect external and internal appearance, it significantly reduces pomegranate fruit marketability. Several postharvest treatments have been proposed to prevent CI, including atmospheric modifications (MA), heat treatments (HT), coatings, use of polyamines (PAs), salicylic acid (SA), jasmonates (JA), melatonin and glycine betaine (GB), among others. There is no complete understanding of the etiology and biochemistry of CI, however, a hypothetical model proposed herein indicates that oxidative stress plays a key role, which alters cell membrane functionality and integrity and alters protein/enzyme biosynthesis associated with chilling injury symptoms. This review discusses the hypothesized mechanism of CI based on recent research, its association to postharvest treatments, and their possible targets. It also indicates that the proposed mode of action model can be used to combine treatments in a hurdle synergistic or additive approach or as the basis for novel technological developments.

## 1. Introduction

**Figure 1:**
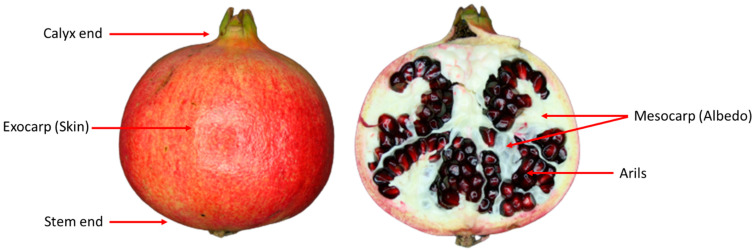
Structure of a typical pomegranate fruit variety ‘Wonderful’ right after harvest including external structures (i.e., calix end, stem end, and exocarp tissue or skin) and internal structures (e.g., mesocarp tissue or albedo and arils or seeds).

*Pomegranate fruit characteristics.* One of the oldest known edible fruits is the pomegranate (*Punica granatum* L.). It is believed to have originated in Iran around 2000 B.C., but was quickly spread throughout the Mediterranean and northern Turkey [1]. Pomegranate is currently grown in Iran, India, Egypt, Lebanon, China, Spain, France, the United States, Oman, Syria, Tunisia, Italy, Greece, Cyprus, Israel, Turkey, Chile, Portugal, and, most recently, South Africa [2]. According to the FAO, pomegranate production worldwide is estimated to be around five million tons. Statistically, the main countries are Iran and India which produce the majority of pomegranates (500,000 tons), followed by China, Turkey, and the United States [3,4]. The fruit is ~5–12 cm in diameter and weighting ~200–650 g consists of an albedo, septa, membranes, and a thick, leathery outer layer, surrounded by a transparent sac that holds 80% of the juice and 20% of the seed (~600 arils) as shown in Figure 1 [5,6].

There are ~153 phytochemicals in pomegranates including phenolic compounds, organic acids, alkaloids, and lignans. In addition to the fruit, other parts of the tree also contain polyphenols. Polyphenols in pomegranate peel and juice include anthocyanins (e.g., delphinidin 3,5-diglucoside, delphinidin 3-glucoside, cyanidin 3,5-diglucoside, cyanidin 3-glucoside, pelargonidin 3,5-diglucoside, and pelargonidin 3-glucoside), other flavonoids (e.g., flavonols such as catechin, epicatechin, gallocatechin), phenolic acids (e.g., gallic, ellagic, caffeic acid), and condensed tannins, while hydrolyzable tannins (e.g., gallotannins, ellagitannins such as pulicalagin and punicalin) are mainly present in peel and membranes. In addition to their red color, anthocyanins have a number of benefits [7,8,9]. According to reports, polyphenols with strong antioxidant and other biological activities also have potent antimutagenic, antihypertensive, and anti-inflammatory actions [10,11,12]. The limited duration of the harvesting season in autumn for only three months greatly restricts the availability and consumption of pomegranate fruit on the market. However, it is possible to store pomegranates for several months in cold, humid storage [13].

**Figure 2:**
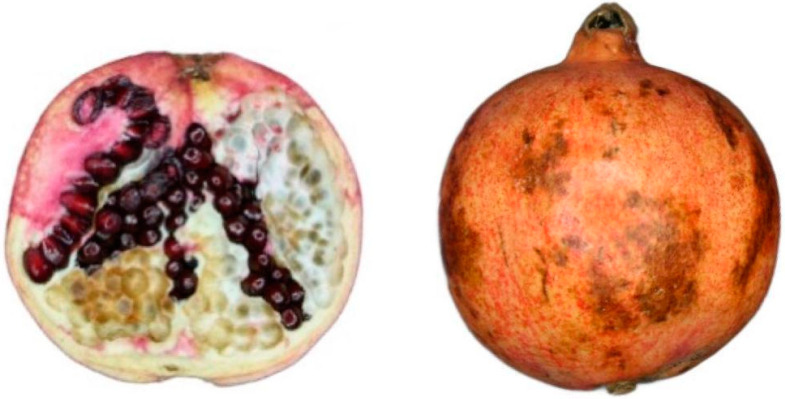
Chilling injury symptoms in pomegranate ‘Wonderful’ fruit including external skin discoloration, and internal albedo browning and aril color changes from red to brown after 120 days of storage at 3.5 °C and ≥95% RH.

*Chilling injury and quality in pomegranate fruit*. Some tropical and subtropical fruit, such as pomegranates, are not recommended for cold storage ≤ 5 °C preservation due to chilling injury (CI) disorder. CI is characterized by pitting and dark brown spots on the whole fruit rind. The internal symptoms manifest as brown discoloration of the white segments between the arils and red color reduction or browning of arils as shown in Figure 2 [14,15,16]. Usually, pomegranates in short-term storage at <5 °C show CI symptoms starting from 6–8 weeks [17,18].

**Figure 3:**
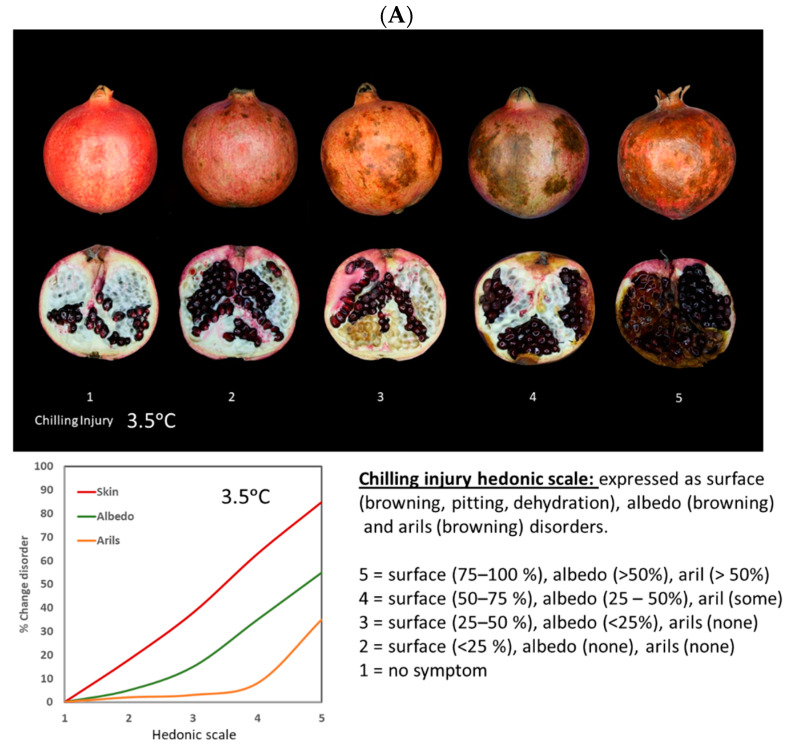

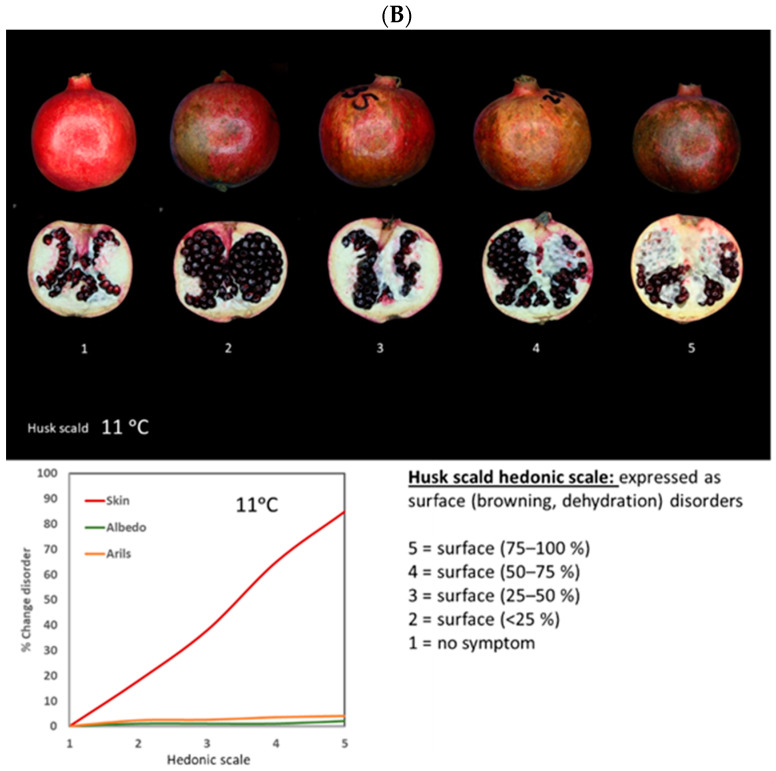
Comparison of quality changes during chilling injury (CI) and husk scald (HS) in ‘Wonderful’ pomegranate fruit. (**A**) Numeric 1–5 hedonic scale for CI in pomegranates stored at 3.5 °C developed within a period of 120 days including visual appearance changes in skin, albedo, and arils. (**B**) Numeric 1–5 hedonic scale for HS in pomegranates stored at 11 °C developed within a period of 120 days including visual appearance changes in skin, with no effects in albedo and arils.

It is widely accepted that damage to the membrane is the trigger for CI. Membranes serve as barriers, preventing damage to the cell and disruption of intracellular processes. Cold stress disrupts multiple metabolic pathways, resulting in ion leakage, energy deficiency, and an excess of reactive oxygen spices (ROS). Eventually, this leads to membrane breakdown and cell death [19,20]. Therefore, the cellular compartments are disrupted, and polyphenol oxidase (PPO) oxidizes tannins and other phenolic compounds, resulting in browning of the skin of pomegranates [14,21,22] and likely a similar mode of action for browning of albedo and arils. Furthermore, the rate of browning development in CI of pomegranate fruit differs among tissues with skin > albedo > arils. For instance, in a hedonic scale used for CI in pomegranates stored at 3.5 °C, these browning developments will differ visually among skin, albedo, and arils, and those % changes can be clearly described for each numeric 1–5 hedonic scale used as shown in Figure 3A. At the highest numeric hedonic scale, fruit susceptibility to fungal growth increases, while pitting may take place at different hedonic scales and is consider the result of surface depression due to cell death of the underlying tissue.

CI is different than husk scald development in pomegranate fruit that usually takes place at temperatures >6–10 °C and appears during long-term storage after 10–12 weeks, and is characterized by skin discoloration due to water loss and mediated by ABA signaling [17,18]. A similar mode of action of browning takes place in husk scald skin browning involving membrane damage, PPO, and oxidation of polyphenols [18]. However, in husk scald there is no visual browning of albedo and arils as shown in Figure 3B for stored pomegranate fruit at 11 °C. Furthermore, a numeric 1–5 hedonic scale for husk scald only considers % changes of skin browning with no effects in albedo and arils.

Consumer satisfaction and repeated purchases are strongly influenced by the visual quality and color of the fruit. Therefore, CI reduces the marketability of fruit and the profitability of producers.

Pomegranates̓ CI has been described in several studies; however, it remains the most significant phenomenon associated with its cold storage. The principal techniques and treatments for alleviating CI, their advantages and disadvantages, along with a hypothetical model of the mode of action involved are discussed in this paper. Our objective is to provide a comprehensive guideline for future research that will help bridge the scientific gap between fruit producers and researchers.

## 2. Hypothetical Model of Fresh Produce Chilling Injury

**Figure 4:**
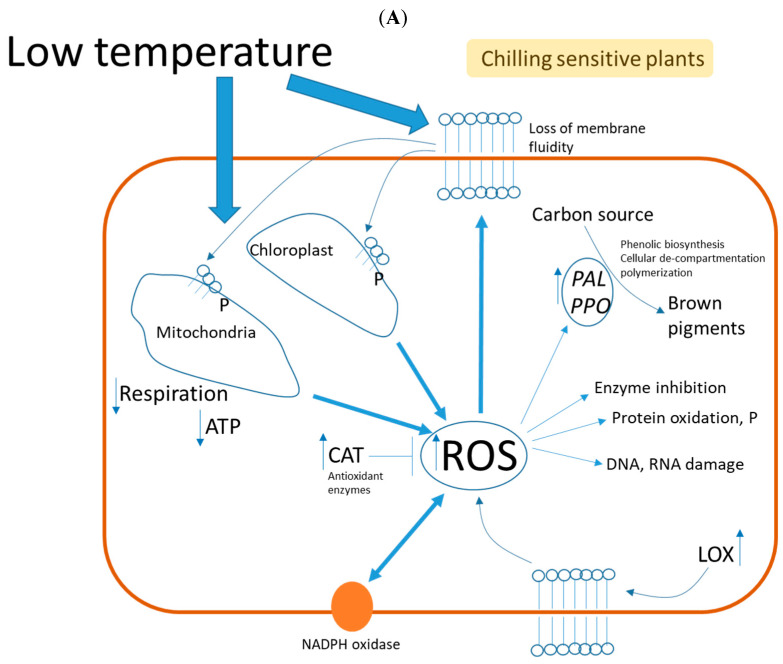

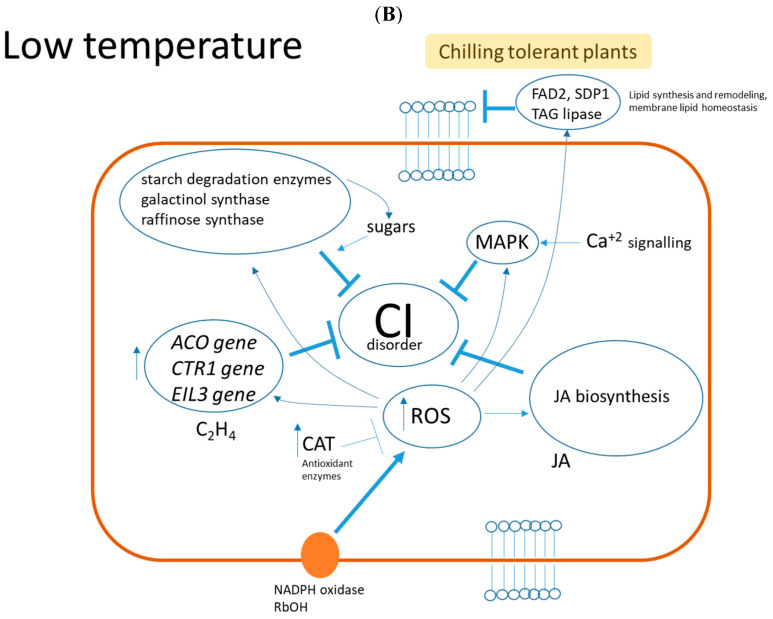
Proposed models of chilling sensitive and chilling tolerant plants based on a dual role mode of action of oxidative stress. (**A**) In chilling sensitive plants, low temperature storage induces higher levels of oxidative stress (e.g., ROS source from chloroplast, mitochondrial and NADPH oxidase activity) and less membrane fluidity. This in turn further damages membrane functionality, induces DNA and RNA damage, alters protein functionality, and enhances phenolic biosynthesis and polymerization, while the intrinsic antioxidant system is overwhelmed by ROS levels causing CI symptoms. (**B**) In chilling tolerant plants, low temperature storage induces several protective mechanisms against CI including induced ethylene and jasmonic acid biosynthesis, released sugar levels are induced, membrane protective mechanisms, induced Ca^+2^ and MAPK signaling, and an enhanced antioxidant system which attenuates and maintains low oxidative stress levels induced mainly by NADPH oxidase. These low ROS levels signal the above protective mechanisms in tolerant plants against CI. CAT, catalase, C_2_H_4_, ethylene, JA, jasmonic acid, CI, chilling injury, ROS, reactive oxygen species, PAL, phenylalanine ammonia lyase, PPO, polyphenol oxidase, LOX, lipoxygenase. Proposed hypothetical models are based on references from Section 2.

Horticultural crops can be stored at low temperatures after harvest, in order to decrease the cell metabolism and delay plant senescence. However, some tropical and subtropical fruits and vegetables are not suitable for this type of storage since they develop physiological disorders. This set of alterations is commonly known as CI. For chilling sensitive subtropical plant species, CI occurs at temperatures around 8 °C, and for tropical plant species, it occurs around 12 °C [23,24,25]. In most commercial pomegranate fruit, chilling injury happens at < 5–7 °C depending on the cultivar [26]. There are a number of microscopic and macroscopic changes that occur following a CI [27]. A change in bio-membrane conformation and structure is considered to be the first molecular event that affects membrane permeability during CI [25,28]. A number of modifications occur, including lipid peroxidation and an increase in the fatty acid saturation index, degradation of phospholipids, and an increase in the sterol/phospholipid ratio. All of these changes in membrane lipid composition lead to a decrease in fluidity, resulting in a decrease in membrane functionality [29,30]. A dysfunctional membrane leads to secondary physiological responses, such as loss of turgor, electrolyte leakage (EL), metabolic energy loss, and a disintegration of photosynthesis [25,31] (Figure 4A).

The second effect of low-temperature stress is an increase in ROS. An excessive abundance of ROS generated by oxidative stress will result in cell oxidative death as lipid peroxidation, protein oxidation, enzymatic inhibition, and DNA and RNA damage take place [32]. Furthermore, CI decouples the electron transport chain in mitochondria and chloroplasts. The membrane proteins involved in electron transference undergo changes that result in an excess production of ROS [33,34,35]. On the other hand, as a result of chilling temperatures, mitochondrial respiration is inhibited, as well as membrane-bound ATPase activity, which causes ATP production to decline sharply [36] (Figure 4A).

The production of ROS can also be greatly enhanced by activating NADPH, which is located in the cell membrane [37,38]. This process leads to the peroxidation of membrane lipids [39]. There is an association between phospholipid catabolism and an increase in the activity of lipoxygenase (LOX) in different plant species during incidence of CI [40,41]. In plants, ROS are scavenged by a sophisticated antioxidant defense system that includes both non-enzymatic and enzymatic components. In addition to polyphenols, ascorbate, glutathione, α-tocopherol, and β-carotene, antioxidant enzymes include superoxide dismutase (SOD), catalase (CAT), ascorbate peroxidase (APX), and glutathione reductase (GR) [33,42,43]. Antioxidant enzymes such as CAT, peroxidase (POD), and SOD appear to play a significant role in determining a plant’s tolerance or sensitivity to CI. It appears that CAT is the main enzyme of the plant antioxidant system that is activated in response to oxidative stress caused by CI [44,45] (Figure 4A).

Two kinds of polyphenols are present in pomegranates including anthocyanins, the main pigments, and hydrolyzable tannins, which are produced from intermediates in the shikimate pathway [46,47]. Antioxidants such as these compounds act as protection against chilling damage [48,49]. However, it has been reported that CI increases the activity of phenylalanine ammonia-lyase (PAL), PPO, and POD in pomegranates, which results in the oxidation of tannins to produce brown compounds [21,50,51]. Pomegranates with CI have been reported to contain high levels of total phenol, PAL activity and gene expression, and low levels of anthocyanin. Cold storage increased PPO activity and gene expression in all tissues tested, regardless of their susceptibility to CI development [22,52] (Figure 4A).

A few studies have been conducted in non-chilling sensitive fruit or chilling tolerant fruit (Figure 3B). The transcriptome responses to cold storage of relatively chilling tolerant ‘Wonderful’ pomegranate fruit and chilling sensitive ‘Ganesh’ pomegranate fruit were compared in a study. Several regulatory (jasmonate, calcium, and MAPK signaling and TFs), metabolic (carbohydrates, phenols, and phenylpropanoids), and stress adaptation (HSPs) transcripts were specifically induced in ‘Wonderful’ fruit in response to cold storage and may thus be involved in conferring chilling tolerance to this variety [53]. The results of the research demonstrated the upregulation of transcripts involved in the biosynthesis and signaling of jasmonic acid (JA) and ethylene hormones [54]. In response to cold stress, ethylene is thought to enhance chilling tolerance, but contradictory evidence suggests it rather increases damage [55,56,57]. On the other hand, it has been reported that the ethylene biosynthesis enzyme ACO as well as the ethylene signaling genes CTR1 (*Pgr019186*) and EIL3 (*Pgr026576*) were up-regulated in the chilling-tolerant fruit, suggesting that ethylene contributes to chilling tolerance [54]. Pomegranates, which are relatively chilling-tolerant, exhibit an upregulation of stress-related transcription factors, including MAPK and Ca^2+^ signaling. Abiotic stress increases the levels of calcium in the cytosol, acting as a second messenger. Ca^2+^ sensors detect the signal, which is transferred downstream to activate the MAPK signaling cascade, resulting in cold tolerance [58,59]. Transcripts for starch degradation enzymes, galactinol synthase, and raffinose synthase are up-regulated in tolerant pomegranates [54]. Cold hardiness is associated with increased degradation of starch into soluble sugars; the accumulation of free sugars provides osmo-protectant for cells [60]. The amount of galactinol and raffinose increases during cold acclimation, according to previous reports [61,62]. Transcripts involved in lipid synthesis and remodeling reported differential expression. FAD_2_ (delta 12 fatty acid desaturase 2), a membrane-modifying enzyme that increases membrane unsaturated fatty acids and adapts to low temperatures by altering its acyl chain, is one of them. Sugar-dependent1 (SDP1), which encodes a triacylglycerol (TAG) lipase involved in directing free fatty acids towards oxidation and so contributes in the maintenance of membrane lipid homeostasis, was also shown to be highly up-regulated in relatively chilling-tolerant pomegranate fruits [54,63,64]. Stress-related transcripts, such as heat shock proteins (HSPs)/chaperones, and oxidative stress transcripts, such as RbOH and NADPH-oxidase, that act as a producer of ROS, whose accumulation can trigger and regulate cellular signaling pathways in response to abiotic stress factors, including cold stress, were also abundant in cold-tolerant pomegranates [54,65,66] (Figure 4B).

According to the above-described mode of actions in chilling sensitive and tolerant fruit, we hypothesize that ROS plays a dual role in these two scenarios. On one side, chilling tolerant fruit have low levels of ROS that signal a mechanism of protection against CI, with those levels of ROS finely tuned by a robust antioxidant system. In chilling sensitive fruit, higher ROS levels damage different biochemical pathways and cell molecular components leading to CI symptoms, where ROS levels overwhelm the antioxidant system present in the fruit. Previously, Cisneros-Zevallos and Jacobo-Velazquez (2021) proposed a model where oxidative stress plays a key role in plants from homeostasis through hormesis to extreme stresses in relation to physiological and molecular responses and quality changes [67] (Figure 4). The above proposed models for CI sensitive and tolerant fruit will be used to discuss the influence of pre-harvest and harvest factors and postharvest treatments for controlling chilling injury in pomegranate fruit.

## 3. Factors Affecting Chilling Injury Incidence

### 3.1. Pre-Harvest Factors

Pre-harvest factors are rarely studied in relation to CI incidence in pomegranate fruits. When it comes to CI, there are significant pomegranate varietal differences. For instance, the ‘Ganesh’ cultivar is more susceptible than ‘Wonderful’ [53]. Furthermore, it is known that the cultivar ‘Wonderful’ is more sensitive to the appearance of CI (7 °C) than the cultivar ‘Mollar de Elche’ (5 °C) [26].

In addition, several pre-harvest treatments have shown to affect quality and CI symptoms of pomegranate fruit during storage. For instance, spraying pomegranates with 1 or 2 mM Methyl jasmonate (MeJA) prior to commercial harvesting improved the aril color at harvest and reduced the postharvest CI index. With storage, EL increased, but it was significantly higher in the control group than in the treated group. Furthermore, MeJA treatments significantly increased flavonoids, total antioxidant activity (TAA), total phenolics (TP), and total anthocyanins as compared to untreated fruits [68]. Despite several studies on this topic, MeJA’s mechanism for reducing CI is not fully understood. Wang and Buta (1994) reported that MeJA could delay chilling by inducing polyamines (PAs) and abscisic acid (ABA) synthesis [69]. Another possible mechanism is the elevation of antioxidant systems [70,71].

Pre-harvest applications of salicylic acid (SA) foliar spray has shown to increase polyphenols, anthocyanin, vitamin C, antioxidant levels, soluble solids, aril color, and fruit yield of ‘Mollar de Elche’ pomegranate fruit [72]. Furthermore, various concentrations of ascorbic acid (ASA) and salicylic acid (SA) were administered three times to Manfalouty pomegranate trees to determine whether they had an impact on fruit quality and shelf life. It was found that SA, alone or in combination with ASA, was effective at preventing fruit CI and preserving fruit quality. It was determined that SA 250 ppm and ASA 250 ppm were the most effective combination [73]. An antioxidant molecule such as ASA plays a vital role in the detoxification of ROS, which may protect plants from environmental stresses such as heavy metals, heat, and salinity [74].

In agriculture, crops are frequently protected from excessive solar radiation by nets and screens made of cloth or other materials in order to prevent heat and light stress [75]. During approximately four months, researchers compared the quality of pomegranate fruit grown in an open field to fruit grown under green 50% net shade. As a result of shade treatment, CI symptoms were reduced, PPO and POD levels were decreased, SOD and CAT levels were elevated, and antioxidant levels, phenolics, and anthocyanins were higher [76]. Pomegranate fruit treated with 1 mM arginine by pre-harvest spray in combination with postharvest immersion had lower CI symptoms as a result of higher antioxidant enzyme activities, such as SOD, CAT, and APX. A greater ratio of PAL/PPO enzyme activity was observed in arginine-treated pomegranate fruit, resulting in a reduction in husk browning. Arils from pomegranate fruits treated with arginine contained higher levels of anthocyanins and total phenols, which contributed to their ability to scavenge DPPH [77].

Deficit irrigation of pomegranate fruit (32% less than the reference evapotranspiration) resulted in increased peel redness and hardness, as well as higher soluble solid content, vitamin C, and total antioxidant capacity (TAC). Moreover, deficiency irrigation delayed the development of CI symptoms, which manifested after 60 days of storage compared with 30 days for the controls [78].

### 3.2. Harvesting Factors

According to postharvest physiology, most fruits proceed through three stages: maturation, ripening, and senescence. Fruit maturity at harvest determines the shelf life of the fruit and its final quality. It is also an indication that the product is ready for harvest. In terms of size, it is fully developed, although it may not be ready for consumption. When a fruit has reached ripeness, it can be considered edible. The process of ripening follows or overlaps that of maturation. A fruit’s senescence is characterized by a loss in texture and flavor as a result of natural degradation. Pomegranates can be harvested when they reach a certain size and skin color. Other maturity indexes include TSS and TA [79]. When it comes to the timing of harvest, whether for immediate fresh market or storage, the timing is critical if the fruit is to reach the consumer in prime condition. Pomegranate fruit maturity indices have been shown to be affected by cultivar differences, growing regions, and maturity status [80,81]. A pomegranate harvested at an early stage of maturity may have a good appearance and be able to withstand postharvest handling, but it may have poor aril color intensity and an undesirable taste. In contrast, a fruit harvested at late maturity is more vulnerable to spoilage and has a shorter shelf life [82]. Research has shown that early-harvested fruits are very susceptible to chilling and should not be stored at low temperatures for extended periods of time. Mid-harvested and late-harvested fruits, however, are more resilient to chilling and will thus retain their quality for longer periods of time, as well as being more suitable for cold quarantine treatment disinfestation [83].

An analysis of transcription factors involved in different harvesting times indicates that transcripts that are associated with multiple regulatory, metabolic, and stress adaptation pathways are particularly upregulated in late-harvested fruit while being downregulated in early-harvested fruit, which is sensitive to chilling. These regulatory mechanisms included the activation of JA and ethylene biosynthesis and signal transduction pathways, as well as the expression of numerous stress-related transcription factors, such as AP2/ERFs, MYBs, WRKYs, bHLH, homeobox, and HSFs. A number of genes related to starch degradation, primary and secondary carbohydrate metabolism, as well as galactinol and raffinose synthesis, were altered. Furthermore, transcripts encoding genes associated with stress tolerance, particularly HSPs, were observed to be more expressed [84]. Another research study examined the gene expression of mature and premature pomegranate fruits. According to the findings, the CI premature fruit had a high total phenol content, which is associated with a high level of antioxidants. The concentration of punicalagin was the same for premature and mature skin at harvest and during storage, and thus was not associated with CI development in the premature fruit skin. Furthermore, the antioxidant-related genes CAT2, SOD, and GR2 were expressed similarly in both immature and mature fruit skin. There was no link found between the levels of expression of antioxidation-related genes and CI susceptibility. According to the findings, high total phenol content, relatively high PAL activity/gene expression, and low anthocyanin levels are associated with higher skin susceptibility to CI. Even though PPO activity and gene expression increased during cold storage, the same trend was evident in all tissues studied, regardless of susceptibility to CI development. A high ratio of PAL to PPO expression was found in mature skin, which may be associated with reduced CI disorder [22,52]. In other studies, it was reported that light colored fruit were more sensitive to CI and husk scald, regardless of having high antioxidant activity [49].

**Figure 5:**
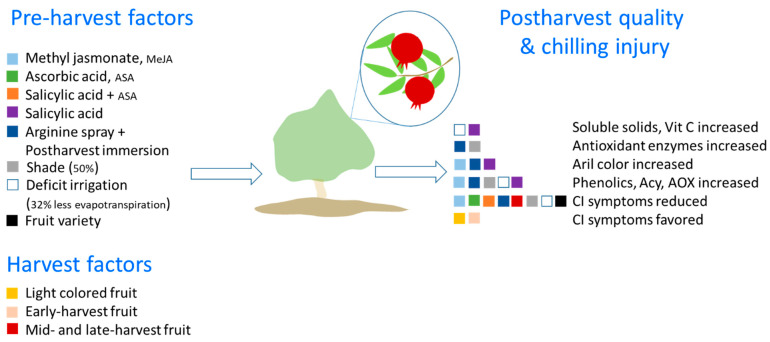
Integral model linking pre-harvest and harvest factors and its effects on chilling injury (CI) development during postharvest storage of pomegranate fruit. This integrative approach based on studies reported in the literature (see Section 3.1 and Section 3.2) can be used as a reference for identifying and introducing future studies on pre-harvest and harvest factors and also dissect their effects on different pomegranate fruit quality attributes and physiological disorders besides CI (e.g., husk scald effects [17]. Accordingly, chemical treatments (e.g., MeJa, ASA, salicylic acid, arginine), fruit variety, fruit maturity, shade, and irrigation will affect quality attributes (e.g., soluble solids, aril color, antioxidant enzymes, polyphenols) and reduce or favor chilling injury symptoms. We hypothesize the pre-harvest factors that prevent CI in pomegranate fruit are strengthening the intrinsic antioxidant system of the fruit and maintaining the stability and functionality of the cell membranes in agreement with CI models in Figure 4.

In Figure 5, an integral model based on the above data linking pre-harvest and harvest factors and its effects on quality and CI development during storage is proposed. This integrative approach can be used to identify and introduce future studies on pre- and harvest factors and to dissect their effects on different pomegranate fruit quality attributes and physiological disorders such as CI effects. According to the hypothetical models of chilling sensitive and tolerant plants (Figure 4), it is likely that these pre-harvest factors that prevent CI in pomegranate fruit are strengthening the intrinsic antioxidant system of the fruit and maintaining the stability and functionality of the cell membranes.

### 3.3. Postharvest Treatments for Controlling Chilling Injury

In Table 1, different postharvest treatments and their effects on controlling CI incidence in pomegranate fruit are summarized, including the use of CA, MAP, film wrapping, heat treatments, coatings, exposure to SA, JA, PAs, and other treatments including the use of melatonin, glycine betaine, and NO. In addition, in Figure 5, we present the possible targets of these postharvest treatments based on a model of CI development proposed herein.

#### 3.3.1. Heat-Treatment (HT)

Due to consumer concern about chemical residues in fruit and vegetables, it is essential to develop safe and environmentally acceptable methods to eliminate or reduce the presence of CI. A significant study has already been conducted in this area of postharvest research. HT (38–60 °C) have become increasingly popular as an environmentally friendly method of reducing CI [85]. Intermittent warming (IW), hot air, vapor heat, and hot water (HW) are all examples of possible HT applications [86]. HT have been demonstrated to effectively reduce low-temperature storage damage when applied to fresh produce before cold storage [87]. Several factors may contribute to the mitigation of chilling in heat-treated fruits and vegetables. (1) Improved membrane integrity can be attributed to an increase in unsaturated fatty acid/saturated fatty acid ratios; (2) improved expression and accumulation of HSPs; (3) improved antioxidant system performance; (4) Improve chilling tolerance by increasing arginine pathways, which result in more signaling molecules such as PAs, nitric oxide, and proline; (5) Changing the activities of the enzymes PAL and PPO; (6) Enhancing the metabolism of sugar [88].

Low-temperature-conditioning (LTC) of pomegranates at 15 °C for 10 days improved chilling tolerance. Based on the study on transcription factors, LTC significantly diminished the drastic changes in gene expression patterns caused by the cold storage, and it activated the regulatory and stress-adaptation processes that may be responsible for enhancing chilling tolerance. LTC elevated ICE-1, a master regulator of cold tolerance, and influenced the responses to two essential phytohormones, ABA and ethylene, both involved in stress responses and cold tolerance acquisition. Furthermore, LTC enhanced HSP transcript levels, which operate as molecular chaperones to protect cell components, primarily proteins, and to maintain cellular homoeostasis under stress circumstances [89]. Furthermore, chilling tolerance by LTC has also been associated with the enhancement of sugar metabolism [49].

Studies found that dipping pomegranates in HW at 45 °C, then storing them at 2 °C, lowered CI. During storage, the HT resulted in an increase in free putrescine (Put) and spermidine (Spd). The higher polyamine levels and unsaturated/saturated fatty acid ratio during storage may maintain membrane integrity and fluidity [90,91].

Pomegranates of the ‘Malas Yazdi’ variety were immersed in HW 45 °C which greatly decreased CI, electrolyte, and K+ leakage, but had no influence on the total soluble solids, total acidity, ASA, and pH of fruit after removal from storage [92].

IW occurs when stored fruit is subjected to temperatures between 20 and 27 °C from time to time [93,94]. It is necessary to know the optimal temperature, duration, and frequency for each product and cultivar. The timing of treatment is essential; cold storage must be interrupted to avoid the irreversible effects of chilling injuries [27,95]. For instance, pomegranate fruit were placed through two different IW cycles of 3 days at 5 °C, then 1 day at 20 °C (IW 3d), and 6 days at 5 °C, then 1 day at 20 °C (IW 6d), with control fruit kept at 5 °C with 90–95% RH. After 3 months of storage, pomegranates with IW had lower WL, lower respiration rate (RR), mainly in the first month, as well as less deterioration, and less fruit loss. However IW treated fruits had a higher incidence of CI than control fruits [96]. In contrast, other studies show successful effects of IW against CI. For instance, during cold storage of pomegranate ‘Rabab-e-Neyriz’ (70 days at 2 ± 0.5 °C and 90 ± 5% RH), IW in the form of a single warming period has been investigated. To determine the appropriate treatment time, warming was conducted at four storage interruption points (after 15, 25, 35, or 45 days). As a result of IW on the 15th day, unsaturated fatty acids were better preserved from peroxidation, malondialdehyde (MDA) production was decreased, and unsaturated/saturated fatty acid ratios (membrane integrity index) were preserved in the peel during storage and chilled injury symptoms were reduced. Furthermore, the levels of spermine (Sp) and Put (important antioxidants functioning as membrane safety agents) increased immediately after treatment and remained higher than the control throughout storage [95]. Furthermore, IW on the 15th day of storage promoted higher enzymatic antioxidant activity and phenolic compounds as well as lowering PPO activity in the peel [97]. The ‘Mollar’ pomegranates were stored at 0 or 5 °C, and every 6 days, IW was applied for one day at 20 °C. It was found that IW at 0 °C maintained the harvest-red color of the skin, decay was prevented but chilling injuries such as pitting were raised, while storage at 5 °C reduced these injuries but did not reduce fungal infection [98]. In other studies, pomegranates were expose at 33 °C and 95% RH for 3 days before being stored at 2 or 5 °C for 90 days. This was compared to cycles of IW of 1 day at 20 °C every 6 days with storage at 2 or 5 °C. The highest anthocyanin contents and titratable acidity were found in IW fruits, as well as the best visual appearance. Meanwhile, IW stored at 2 °C was the only treatment that produced fruit with a flavor similar to that of harvest. IW at 2 °C has proven to be the most effective treatment for preventing chilling damage and preserving pomegranate fruit quality [99].

As a result of conditioning pomegranate fruits at 38 °C for 24 and 36 h and 1.5 °C storage, CI signs (browning), as well as weight loss (WL), were significantly reduced, while EL, total soluble solids, total acidity, ASA, and pH were not affected by conditioning. Fruit juice qualities were unaltered by conditioning at 55 °C for 30, 60, 90, and 120 min. However, chilling damage symptoms, EL, and potassium leakage were reduced [100].

**Figure 6:**
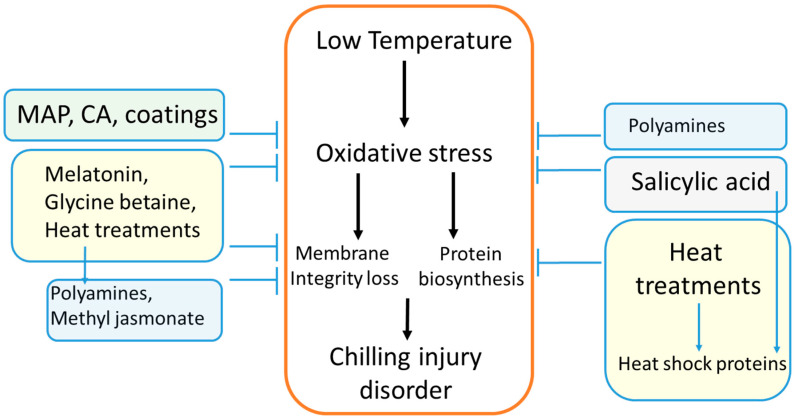
Hypothesis of pomegranate chilling injury (CI) incidence during long-term storage and the possible targets of postharvest treatments. Accordingly, CA, MAP, and coating systems would work mainly by decreasing oxidative stress by reducing the presence of internal oxygen within the fruit. On the other hand, heat treatments may exert their effects by inducing heat shock proteins in a hierarchical stress response model or alternatively by reducing oxidative stress and preserving membrane fluidity and by inducing polyamines. Furthermore, applied polyamines, melatonin, and glycine betaine may have a dual role of reducing oxidative stress and preserving membrane integrity. SA attenuates oxidative stress and induces heat shock-like proteins while methyl jasmonate preserves membrane integrity. Proposed hypothetical model is based on references from Section 3.3. Proposed model could be the basis of a hurdle strategy combining different technologies to attenuate CI in a similar fashion as previously described for husk scald disorder in pomegranate fruit [17].

These results suggest that postharvest applied HT protect against CI in pomegranates mainly through ameliorating oxidative stress, reducing membrane integrity loss, and attenuating biosynthesis of proteins involved in CI disorders by inducing HSPsas well as by inducing PA synthesis (Figure 6).

#### 3.3.2. Polyamines (PAs)

A polyamine (PAs) is a positively charged aliphatic amine found in living organisms that has been implicated in a number of biological processes including plant growth, development, and stress response, Put, Spd, and Sp are examples of common PA [101].

PAs inhibit several senescence-related processes in a variety of plant species, as well as acting as free radical scavengers [102,103]. It has been shown that PAs may interact with anionic components of membranes, such as phospholipids and thus stabilize the bilayer surface [104,105]. As well as their ability to interact with phospholipids, PAs could protect membranes from peroxidation. CI causes membrane damage, and PAs protect membranes, so the possible link between PAs and CI is of great interest [102].

During two months of storage, Put (2 mmol/L) reduced the incidence of physiological issues such as exterior deterioration, husk scald, and CI in pomegranates. However, a higher concentration is recommended for longer storage periods, as a 3 mmol/L dosage proved the most efficient at reducing CI and preserving physicochemical properties and sensory qualities throughout storage [106]. Put and Spd were applied for pomegranate fruit prior to storage. Skin browning, EL, WL, losing firmness and color deterioration were dramatically delayed by PA treatments. Increased levels of free endogenous Put and Spd in the epidermis might lead to improved shelf life by enhancing pomegranate adaptation to low temperatures and CI protection [107].

Pomegranate fruits were treated with 2% calcium chloride in combination with 2 mM Spd had significantly higher CAT and SOD activities and lower POD activities [108].

A combination of Put and carnauba wax can reduce the softening of fruit, respiration, and ethylene evolution rate, as well as WL. As a result, pomegranate fruits may have a shelf life exceeding 60 days when stored at low temperatures (3 °C) [109].

Accordingly, reports suggest that postharvest applications of PAs in pomegranates may protect from CI by ameliorating oxidative stress and by maintaining membrane integrity (Figure 6).

#### 3.3.3. Salicylic Acid (SA) and Jasmonate (JA)

Acetyl salicylic acid (ASA), methyl salicylate (MeSA), and MeJA are all derivatives of SA and JA that play crucial roles in plant defense and signaling. Natural, non-toxic phenolic compounds such as SAs and JAs have been proposed for use in enhancing the quality of postharvest horticultural crops [110].

In several chilling-sensitive fruits during storage, SA treatment reduced the CI symptoms [111,112,113]. The effects of SA on postharvest chilling injuries in fruits and vegetables can be mitigated by enhancing the expression of alternative oxidase (AOX) genes such as ROS suppressing genes in tomato fruit [114]. APX and GR activities, and the ratio (AsA/DHAsA) and the ratio (GSSH/GSSG) of oxidized to reduced ascorbate in peach fruit [113], as well as the production of HSPs, are all observed to have an impact [113,115].

In an investigation with pomegranates, SA treatments, particularly at 2 mM concentration, were found to be significantly successful in lowering CI and EL, inhibiting ASA decline, and delaying an increase in PAL activity when compared to controls [111]. A decrease in unsaturated/saturated fatty acid ratio as well as softening and loss of fatty acids were associated with the severity of damage in control fruit. SA’s ability to trigger antioxidant mechanisms and maintain unsaturated fatty acid levels during cold storage may explain its ability to mitigate chilling damage in pomegranates [116].

It is known that JA regulates the responses of plants to biotic and abiotic stresses, including cold stress [117,118]. In pine, rice, and apple, endogenous levels have been shown to increase in response to cold stress [119,120,121]. JA has also been found to improve chilling resistance in various fruits, including pomegranates [122].

In a study of pomegranates treated with 0.1 mmol/L MeJA before storage, MeJA treatment effectively preserved edible quality, suppressed PPO activity and CI index development, and inhibited the decrease in phenol content as well as the increase in malondialdehyde content and EL. The treated fruit had a greater soluble protein (SPC) content than the control fruit. Fruit may create a stress resistance response and synthesize new proteins in response to low temperatures and MeJA. After 90 days, MeJA and control pomegranate pericarps were microstructurally different. The waxy coating and cuticle of the fruit were largely intact as a result of the treatment, and the epidermal outer cells were properly aligned. The control group, on the other hand, demonstrated lateral cell damage, waxy layer loss, cuticle rupture, and epidermal cell loosening [123]. MeJA reduced CI symptoms both internally and externally when used as a pre and pre+ postharvest treatment in pomegranate. Both pre- and post-treatments may prevent peroxidation of cell membrane lipids, resulting in a reduction in CI and improved membrane integrity due to a higher UFA/SFA ratio. Throughout the storage period, arils from control fruit had lower concentrations of TP and anthocyanins than arils from Pre and Pre+Post-treated fruit. CI, membrane stability, and bioactive molecules with antioxidant activity did not differ significantly between Pre and Pre+Post MeJa treatments [124].

Applying MeJA or MeSA at two concentrations (0.01 and 0.1 mM) was compared in a research study. MeJA or MeSA treatments significantly reduced the symptoms of CI, with no significant differences between treatments or doses. In comparison to controls, both treatments significantly increased TP and anthocyanins. Both hydrophilic (H-TAA) and lipophilic (L-TAA) increased in MeJA or MeSA-treated fruit compared to control arils [125].

In general, these reports suggest that postharvest applications of SA may protect pomegranates from CI by ameliorating oxidative stress and by inducing HSPs, while postharvest applications of MeJA may protect from CI by maintaining membrane integrity (Figure 6).

#### 3.3.4. Control Atmosphere (CA) and Modified Atmosphere Packaging (MAP)

Control atmosphere (CA) storage is a combination of low temperatures with reduced oxygen and increased carbon dioxide concentrations, which reduces fruit RR and ethylene production, suppresses or delays senescence processes, and ultimately extends the shelf life of fruits after harvest [126,127]. Modified atmosphere packaging (MAP), a convenient and less expensive alternative to CA, has also been adopted for short-term storage and transportation of a wide variety of fruits on a commercial basis. Low O_2_ conditions appeared to inhibit the production of ROS and their efficient scavenging via the coordinated action of SOD and POD [128].

Various combinations of CA storage were effective for controlling CI in pomegranates. The most successful combination was 5% CO_2_ + 3% O_2_ and 15% CO_2_ + 5% O_2_ [129,130].

Comparing SA, Put, and Oxalic acid treatments on pomegranates held under CA (5% O_2_ + 15% CO_2_) over a period of six months revealed they all slowed sugar loss and improved phenolic and antioxidant activity without any CI symptoms. Conversely, Put displayed superior results in terms of WL, TA, sugar, TP, and antioxidant activity when compared to all other treatments [131]. It was found that superatmospheric oxygen levels of 50 or 97% were ineffective in preventing pomegranate chilling injuries [132].

Pomegranate, packaged in a variety of polymers including high ethylene absorption (HEA), perforated polyethylene (PPE), polyethylene (PE) film, and commercial polyvinyl chloride (PVC), successfully decreases CI independent of the film material [133]. According to research, pomegranates packaged with 85% CO_2_ are less likely to be damaged by chilling [134]. The effects of black seed oil (0.1% and 0.5%), propolis (0.01% and 0.1%), and fludioxonil (0.06%), with and without modified atmosphere packaging (MAP) was studied. Results showed 0.5% black seed oil or 0.1% propolis was effective in controlling gray mold development and delaying the occurrence of CI, especially when combined with MAP [135].

The effects of MAP, 1-MCP alone or in combination, as well as exogenous ethylene treatments and microperforated bags (MPB-control treatment) were studied in an experiment. In MAP systems, 1-MCP, and combined MAP+1-MCP treatments, CI symptoms were delayed by up to 120 days. Control treatments with ethylene and MPB caused considerable EL, lipid peroxidation, higher oxidative damage such as PAL, PPO, CAT, SOD, and POX activity, and lower phenolic compound. A greater saturated/unsaturated fatty acid ratio after 1-MCP treatments protected peel tissues [136].

Accordingly, reported studies suggest that CA and MAP likely reduce CI in pomegranates by reducing the levels of oxidative stress within cells (Figure 6).

#### 3.3.5. Coating

Coatings protect fresh produce while exerting different functions including their usage in food packaging to decrease environmental issues created by non-renewable synthetic material [137], to prevent mechanical damage during shipping, transit, and storage [138], to serve as carriers for beneficial chemicals such as antibacterial compounds, color or scent enhancers, antioxidants, and anti-ripening components [139], and to decrease water loss and modify internal atmospheres [140,141,142].

Pomegranate fruits were coated with carboxymethyl cellulose (CMC, 2% *w*/*v*) and chitosan (CH, 1.5% *w*/*v*) edible coatings alone or combined with oxalic (OA) and malic acids (MA). As a result of cold storage, CI decreased in treated fruit as compared to controls. As a result of combining treatments, EL and MDA were reduced, H_2_O_2_ was lowered, and TPC, AA, and CAT were increased. In contrast, the control had a lower ratio of unsaturated to saturated fatty acids (unSFA/SFA). A 77% difference in CI was obtained in the CH + 5 mM OA, CH + 50 mM MA, and CMC + 50 mM MA treatments when compared to the control [143]. Coating with CH and potassium sorbate (PS), both alone and in combination, were effective in reducing CI symptoms. These treatments result in increased antioxidant capacity, increased PAL activity, and decreased PPO [144]. SA, chitosan coating, and salicyloyl chitosan coating treatments on CI demonstrated Salicyloyl chitosan application reduced pomegranate fruit CI better than SA and chitosan coating alone [145]. The fruit’s tolerance to CI was improved by coating it with γ- aminobutyric acid (GABA) [146]. Except for its indirect influence on improved antioxidant activity, GABA has the capacity to suppress ROS [147].

With the limited information available on the mode of action of coatings against CI in pomegranate fruit, it is possible that coatings attenuate oxidative stress events (Figure 6).

#### 3.3.6. Other Treatments

Melatonin, an indole derivative of tryptophan, regulates photoperiodism, stimulates plant growth, and resists biotic and abiotic stress [148]. Melatonin is a free radical scavenger and antioxidant activator that prevents cell membrane oxidative damage in addition to regulating plant development [149].

Application of melatonin dramatically reduces CI in pomegranates by decreasing PPO activity and increasing PAL, CAT, APX, and SOD. This lowers the activity of membrane degrading enzymes phospholipase D (PLD) and LOX. Melatonin-treated pomegranate fruits may contribute to adequate intracellular NADPH supply by promoting G6PDH and 6PGDH activities during cold storage [150,151,152].

Higher plant chloroplasts synthesize osmotic regulator glycine betaine (GB) from serine, ethanolamine, choline, and betaine aldehyde [153]. GB suppresses membrane phase transitions from liquid crystal to solid-gel under cold stress [154]. In pomegranate, exogenous GB, especially at 20 mM, reduced CI symptoms, increased endogenous GB and proline, and lowered EL and MDA. SOD, CAT, and APX were also activated by this treatment. GB-treated pomegranate fruits had high amounts of total phenols, flavonoids, and anthocyanin due to PAL activity and reduced PPO activity [155].

Nitric oxide (NO), a bioactive molecule with numerous signaling activities in plants, has been shown to delay senescence and ripening in several fruits due to its effects on respiration, ethylene production, disease incidence, peel color, and enzyme activity [156].

In pomegranate fruit application of NO alone or combined with film wrapping significantly increased antioxidant activity and anthocyanin content, while CI and EL decreased, and the impact was greater when NO was combined with film wrapping [157,158]. More work is needed to understand its mode of action.

Oxalic acid, a natural organic anion found in all plants, has many functions in living organisms. Oxalic acid controls fruit tissue browning [159], induces systemic resistance [160], retards fruit ripening, and controls decay [161]. Pomegranate fruits treated with oxalic acid at 6 mM significantly reduced symptoms of CI, while containing higher levels of TP, ASA, and TAA in both hydrophilic and lipophilic fractions [162]. The mode of action of oxalic acid remains unknown.

In general, results indicate that melatonin and glycine betaine may reduce CI in pomegranates by attenuating oxidative stress as well as protecting cell membrane integrity (Figure 6).

**Table 1:** Summary of postharvest treatments applied to prevent CI in pomegranate fruit during storage.

Pomegranate Postharvest Treatments	Treatment Description		Key Findings	References
**Heat Treatments (HT)**	**LTC**	15 °C for 10 days	Improving chilling tolerance by activation of ABA signaling pathway, downregulation of ethylene synthesis and increasing HSP	[89]
	**HW**	45 °C	Decreased CI increase in free Put and Spd	[90,91,92]
	**IW**	cycles of 3 days at 5 °C, then 1 day at 20 °C (IW 3d), and 6 days at 5 °C, then 1 day at 20 °C	Increased CI symptoms but reduced decay significantly	[96]
	**IW**	single warming period (1 day at 20 °C) after 15, 25, 35, or 45 days and stored at 2 °C	Decreased CI, preservation of unsaturated fatty acids, increase in Put and Sp	[95,97]
	**IW**	every 6 days, IW was applied for one day at 20 °C and stored at 2 and 5 °C storage	Decreased CI	[98,99]
	**Conditioning**	at 38 °C for 24 and 36 h or at 55 °C for 30, 60, 90, and 120 min at 1.5 °C storage	Decreased CI	[100]
**Polyamines (Pas)**	**Put**	Put (1, 2 and 3 mmol/L) and 5 °C	Higher concentrations lower CI during longer storage period	[106]
	**Put**	Put or Spd at 1mM under pressure-infiltration or immersion and storage at 2 °C	increasing the levels of endogenous Put and Spd, pressure-infiltration was recommended	[107]
	**Put+ CaCl_2_**	4% CaCl_2_ + 2 mM Spd (Vacuum infiltration) and at 2 °C storage	Decreased CI	[108]
	**Put + carnauba wax**	2 mM Put and at 3 °C storage	Decreased CI	[109]
**Salicylic acid/Jasmonic acid** **(SA/JA)**	**SA**	2 mM and at 3 °C storage	Decreased CI and maintain unsaturated fatty acid levels	[111,116]
	**MeJA+ packeged in PE fresh-keeping bags**	0.1 mmol/L MeJA at 2 °C storage	Decreased CI	[123]
	**MeJA**	5 mM pre- and pre+ postharvest at 2 °C storage	Decreased CI	[124]
	**MeSA or MeJA**	(0.01 and 0.1 mM) at 2 °C storage	Decreased CI without significant difference	[125]
**Controlled atmospheres/Modified atmosphere packaging** **(CA/MAP)**	**CA**	5% CO_2_ + 3% O_2_ and 15% CO_2_ + 5% O_2_ at 5 °C storage	The most successful combination reducing CI	[129,130]
	**5%O_2_ +15% CO_2_**	When combined with 6 mM oxalic acid, 2 mM Put, or 2 mM SA and at 6 °C storage	Decreased CI	[131]
	**Superatmospheric O_2_**	50 or 97% at 7 °C storage	Ineffective on CI	[132]
	**MAP**	HEA, PPE, PE and PVC at 5 °C storage	All decreased CI	[133]
	**MAP**	85% CO_2_ exposure for 12 h at 18 °C, at 5 °C storage	Decreased CI	[134]
	**MAP**	In combination with 0.5% black seed oil or 0.1% propolis at 6.5 °C storage	Decreased CI	[135]
	**MAP**	1-MCP 1 µL/L alone or in combination with MAP, at 2 °C storage	Decreased CI	[136]
**Coating**	**(CMC, 2% *w*/*v*) and (CH, 1.5% *w*/*v*)**	alone or in combination with oxalic acid CH + 5 mM OA, CH + 50 mM malic acid, and CMC + 50 mM malic acid at 2 °C storage	Decreased CI	[143]
	**CH**	With or without PS at 4 °C storage	Decreased CI	[144]
	**Salicyloyl chitosan**	0.57% salicyloyl chitosan at 4 °C storage	Decreased CI, higher (unSFA/SFA) ratio. Higher hydrophilic (H-TAA) and lipophilic (L-TAA) antioxidant capacity	[145]
**Other treatments**	**GABA**	15 mM at 4 °C storage	Decreased CI	[146]
	**Melatonin**	100 µM at 4 °C storage	Decreased CI	[150,151,152]
	**GB**	20 mM at 4 °C storage	Decreased CI	[155]
	**NO**	300 µM + film wrapping at 1 °C storage or 1000 µM at 5 °C storage	Decreased CI	[157,158]

## 4. Conclusions

In pomegranate fruit during long-term storage, CI has a significant negative effect on marketability. The results of this review paper and the hypothetical model of mode of action for CI disorder described herein suggest that storage duration more likely triggers a cascade of oxidative changes that eventually cause the CI symptoms (Figure 4). This process of CI development apparently starts with oxidative stress imbalance and altered membrane functionality. It is interesting that all of the successful postharvest treatments proposed so far in the literature to attenuate CI development in pomegranates can be explained by the proposed model of CI development described herein (Figure 6), either through control of oxidative stress, maintaining membrane integrity or through redirecting of protein synthesis of key proteins/enzymes involved in CI development. In addition, this CI model sets the basis to explain why pre-harvest and harvest factors may attenuate CI symptoms (Figure 5). This review paper on CI development and mode of action can be the basis for revisiting known postharvest treatments to find and develop new techniques that can prevent CI development. For instance, the combination of treatments using a hurdle approach based on mode of actions of individual treatments may allow us to obtain additive or synergistic effects on the reduction in CI incidence. In spite of the fact that there are a number of studies on controlling CI in the literature, proposing a model describing CI etiology and its prevention or even prediction seems essential for developing novel approaches to reduce CI. Further studies could strengthen the model of CI proposed herein.

## Data Availability

The data presented in this study are available on request from the corresponding author.
